# Optimization of P& PI controller parameters for variable speed drive systems using a flower pollination algorithm

**DOI:** 10.1016/j.heliyon.2020.e04648

**Published:** 2020-08-09

**Authors:** Safwan Nadweh, Ola Khaddam, Ghassan Hayek, Bassam Atieh, Hassan Haes Alhelou

**Affiliations:** a Power Electrical Engineering, Tishreen University, Lattakia, Syria; b Mechatronics Engineering, Manara University, Lattakia, Syria

**Keywords:** Electrical engineering, Energy, Industrial engineering, Computer-aided engineering, Variable speed drive systems (VSDS), Four quadrant chopper, Power quality, Flower pollination algorithm (FPA), Optimization algorithms

## Abstract

In this study, a novel flower pollination algorithm (FPA) has been suggested for optimal tuning of P and PI controllers included in a variable speed drive VSD system control circuit. In addition to the manual tuning of controllers’ parameters, several optimization algorithms like GA, PSO, GWO have been used for controllers optimal tuning in different studies. In this study, the errors caused by grid side current harmonic distortion in the VSD system have been used depending on SPWM technique to generate switching signals for a four-quadrant chopper circuit that acts like a filter and compensates the harmonic content in the grid current and smooth ripples in both DC-link current and voltage. The studied drive system and control circuit have been modeled using Matlab/Simulink, then it was linked with the proposed algorithm and tested under different operation conditions. A hardware laboratory model has been built and tested in order to confirm the validity of this study. Comparing the proposed algorithm with other optimization algorithms shows that the suggested algorithm outperforms other algorithms in improving the time response of the VSD system, reducing total harmonic distortion THD of grid current, and reducing ripple factor PF.

## 1 Introduction

Electrical drive systems are one of the mainstays of the national economy today as they form the basis of many applications in modern life. The main core of such systems is electronic devices, which have great potentials manifested in the increased production, reduced costs, high efficiency and optimum investment of energy. The development of integrated, lower-cost drive systems is expected to impact both the grid and the combined motor in the VSD system, so there are some specifications that must be considered when developing such systems. For example, the grid currents must be within certain limits and the currents injected into the machines should not cause overheating. In addition, the electric drive should not cause any kind of disturbance to the other loads connected in the point of common coupling PCC. The performance of the grid side in a VSD system can be evaluated using the value of THD factor of the grid currents. THD is defined using the ratio between the R.M.S value of any particular harmonic H and the R.M.S value of the total current. In addition, the ripple factor RF of both DC-link Currents and voltages effects the overall performance of the VSD system. When studying the performance of grid side in a VSD, it is necessary to study the disturbances in the grid currents in both steady and transient states under different operation conditions. Time response of the VSD grid side can be improved by reducing the grid currents distortions to a tolerable level and smoothing ripples in DC side voltages and currents. In order to reduce the DC-link current ripple factor, a converter must be placed at the diode rectifier bridge output in the VSD system [1]. Different converters schemes were introduced for that purpose. In [2, 3], a boost chopper was used to adjust the Dc-link current. A buck-boost chopper, which is one of the most important converters types, was introduced in [4, 5] to improve the power quality of the grid side. It was also used to smooth both voltage and current waves in Dc-link [6]. These different converters schemes need efficient modulation techniques to generate switching signals for the transistors in these circuits. Many modulation techniques can be used for that purpose, such as pulse width modulation PWM that is considered to be the most widespread modulation technique. In this technique, the motor voltage is changed by modulating the width and polarity of the voltage pulse. These control circuits are responsible for switching signals generation and they may include different types of controllers such as P and PI. The proper selection of these controllers’ parameters affects the performance of control circuit, which in turn affects the performance of VSD system, so an effective optimization technique must be used to detect the optimal values of these parameters instead of using conventional methods such as manual tuning which consume time and effort. Many optimization techniques have been used for that purpose, such as Particle swarm optimization PSO algorithm, and Grey wolf optimization GWO algorithm. Despite the improvements in the performance of the drive system, both THD and RF are still of high values. In order to overcome these demerits, new algorithm is proposed to improve control system and achieve better values and consequently a better performance of the studied VSD system.

In recent years, many researches introduced FPA for solving different optimization problems. The most important research in this field is one concerned in designing a scheme for soft starting in induction motor depending on FPA-based PI controller and the study found that FPA converges faster to provide near-optimal global parameters for PI controller [7].

In this paper, a novel FPA algorithm has been used for finding the optimal parameters of P and PI controllers included in a VSD control circuit. The performed Simulation and experimental study show that this algorithm achieved a better time response of the studied VSD system and reduced Values of the grid current THD and DC-link RF.

Section 2 gives detailed information about studied drive and control system, section 3 explains Genetic Algorithm and Flower Pollination Algorithm in details, Section 4 discusses the proposed method in details, section 5 presents and discusses the results of the studied system, and finally the conclusions drawn from this work are given in Section 6.

## 2 Drive and control system models

The studied system is a VSD system with a hybrid filter (serial-parallel), which is a four-quadrant chopper, integrated in DC-link to enhance the characteristics of the input currents of this system. In order to analyze the performance of the studied VSD system under different operation conditions, a simplified model of this system is required. The model is consisted of a grid, rectifier, filter, inverter and a motor that is replaced by a resistive load in order to simplify the study. The grid is a three-phase ac voltage system which voltages are given by Eqs. (1), (2), and (3) [8]:

(1) Van=Vi.cos(wt)

(2) $$Vbn=Vi.\cos\left(wt-\frac{2.\pi }{3}\right)$$

(3) $$Vcn=Vi.\cos\left(wt-\frac{4.\pi }{3}\right)$$

The diode bridge rectifier output is given by Eqs. (4), (5), and (6) [8]:

(4) Vg=VA−VB

where:

(5) VA=max(Van,Vbn,Vcn)

(6) VB=min(Van,Vbn,Vcn)

The chopper voltage is the difference between the dc-link voltage and the inverter voltage and it can be expressed by Eq. (7) [8]:

(7) Ve=Vc(t)∗(1−2d(t))

*d(t)*: is the operation duration of the switch.

**Figure 1:**
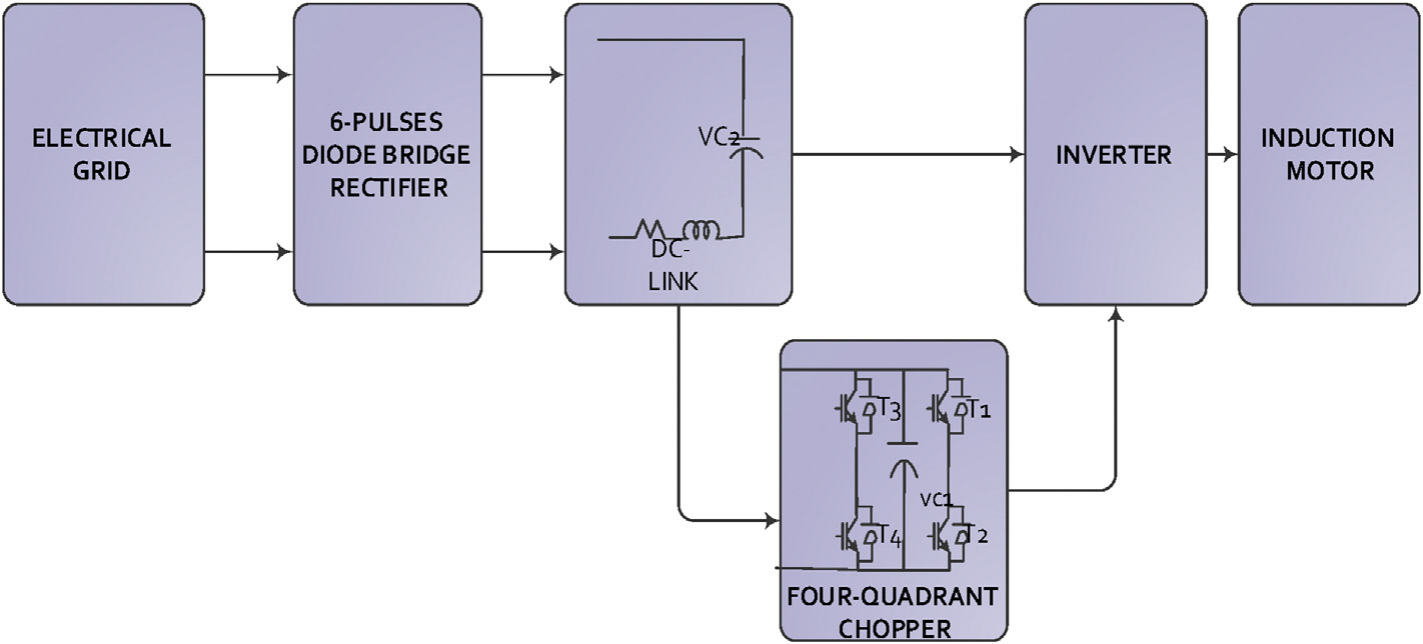
Schematic diagram of Variable Speed Drive System.

**Figure 2:**
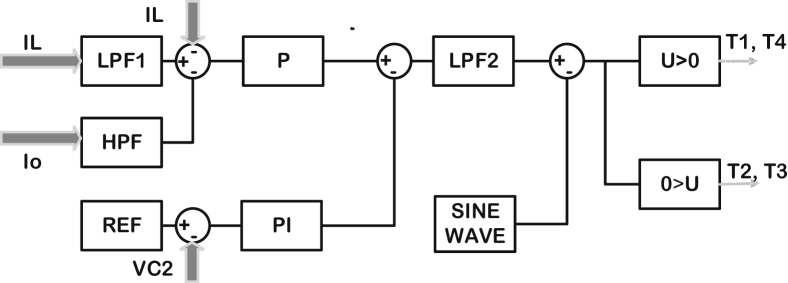
Control circuit model.

The proposed control circuit consists of two parts: the first part includes a P controller that processes the current error, while the second part includes a PI controller that processes DC-link voltage error. The second part also helps to maintain a constant current in the DC-link inductor and to remove fluctuations. The sum of output control signals of both controllers forms the input signal for SPWM control technique to generate switching signals for the filter transistors. In addition, low and high pass filters have been used in this control circuit. Both P and PI controllers have been tuned using FPA depending on the integration of time multiplied by absolute error objective function. When this function reaches its minimum value, the optimal parameters of controllers are obtained. The studied VSD system and control circuit are shown in Figures 1 and 2 respectively.

## 3 Genetic & flower pollination algorithm

Over millions of years, many vital systems evolved greatly by achieving evolutionary goals such as reproduction. Building on the success of vital properties, many algorithms have been created over the past years. For example, genetic algorithm depends on the development of biological systems, particle swarm optimization PSO theory is associated with the manner of crowds of birds and fish. These algorithms have been implemented in different types of applications. In many Engineering and industrial design applications, it is necessary to find the optimal solution for a specific problem under complex conditions. Finding optimal solutions is usually a non-linear process, so it may be very difficult or even impossible to find optimal solutions. Most common optimization techniques do not operate in a good way with non-linear and multi-constrained problems, so the current trend in optimization is toward using naturally inspired algorithms to discuss such problems.

**Figure 3:**
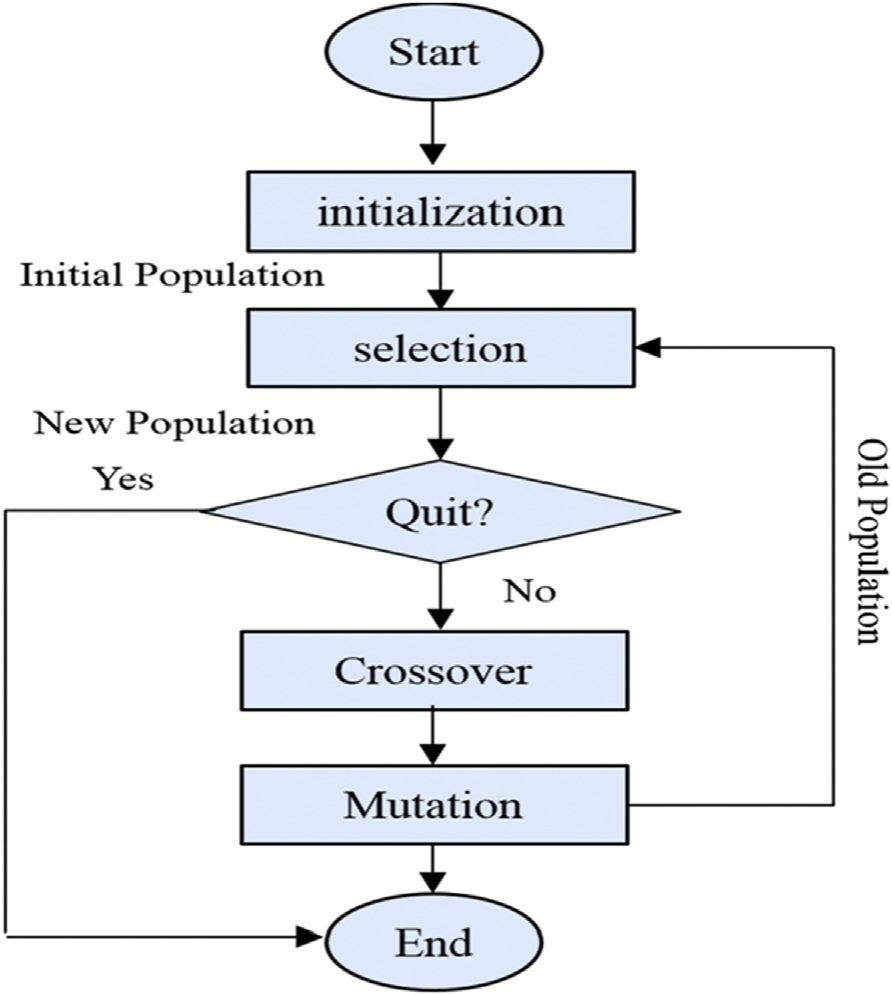
The flow chart of genetic algorithm.

Genetic algorithm is an optimization method that can be used to solve both constrained and unconstrained issues based on natural selection. This algorithm starts by creating a random initial population, then generates a succession of new populations. At each step, the algorithm uses the individuals in the current population to generate the new population, which means genetic algorithm frequently modifies a population of each solution. Genetic algorithm is generally used to create high-quality solutions for optimization and search issues depending on bio-inspired factors such as mutation, crossover, and selection. In addition, it uses probabilistic selection rules instead of deterministic rules. Since this algorithm starts from a population of points, it can avoid locally trapped optimal solutions as in traditional methods that starts search from a single point. The flow chart of GA shows in figure (3).

Pollination of flowers algorithm (FPA) is a new optimization algorithm that is inspired by the flower pollination process. From a biological evolution point of view, the goal of pollination of the flower is the permanence of the fittest and the ideal breed in plants in terms of numbers and preference. The basic idea of flower pollination within the context of bees and populations was studied in previous algorithms, but this algorithm is only based on the pollination characteristics [9].

### 3.1 Characteristics of pollination of the flower

Statistics show that there are more than four million species of plants in nature, about 80% of them are flowering plants. The essential goal of a flower is reproduction by the pollination process. Pollination is mainly related with pollen movement, which is done by pollinators such as insects, birds, bats, and other animals. Some flowers and insects have a mutual evolutionary relationship that develops into a very special reproduction relationship. For example, some kinds of flowers can only entice and exploit a certain kind of insect to complete pollination. Pollination takes two forms: biotic and non-biotic [10]. About 90% of flowering plants are pollinated by bio-pollination. In this type of pollination, pollen is transmitted by pollinators such as insects and other animals.

About 10% of pollination is non-biotic and does not need any pollinator. Wind and water-assisted propagation transmit pollen to the plants in this form of pollination. Pollinators can be very diverse; studies show that there are about 200 000 kinds of pollinators. These pollinators can develop the stability of the flower. The concept of flower pollination can be simplified using the following rules:

1) Biotic and cross-pollination are regarded as a global pollination. In these types of pollination, pollen carriers use levy flight search strategy.
2) Abiotic and self-pollination are used for Local pollination.
3) Pollinators such as birds, insects may develop the flower stability, which rises the probability of reproduction, and that relatively depends on the likeness of the two flowers included in the reproduction operation.
4) The switching between local and global pollination can be controlled by switching probability value that ranges between 0 and 1 with a slight bias towards local pollination.

**Figure 4:**
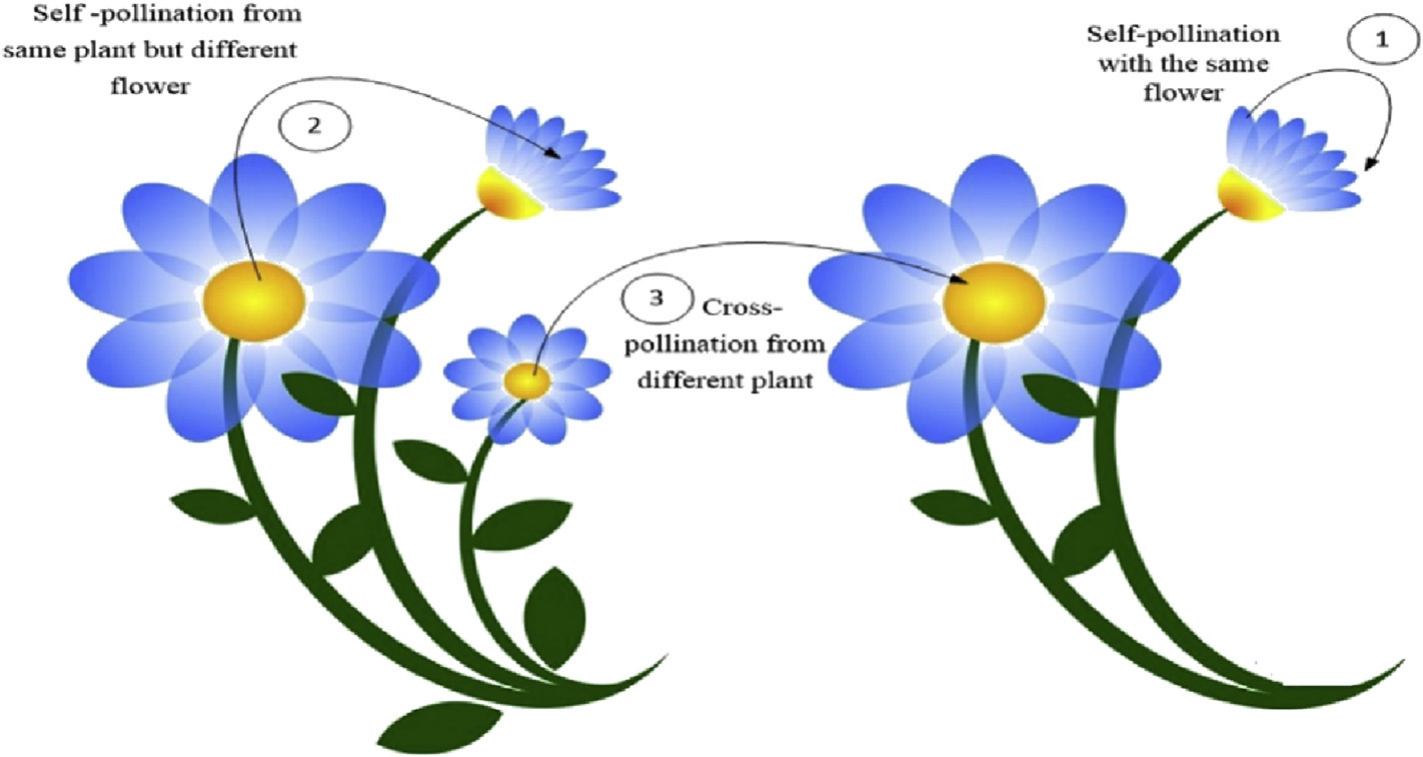
Forms of flower pollination.

In global pollination, pollinators such as birds, winds, etc. carry pollen and transfer it over long distances. The different forms of pollination are shown in figure (4).

### 3.2 Steps of the flower pollination algorithm

Step 1 Initialize a random population of flowers (n), then find the objective function value for each flower and choose a universal flower to the best solution.

Step 2 Generate a random number for each flower, if the number is smaller than switching probability, then draw a vector L with d dimension and apply global pollination as in Eq. (8) [11]:

(8) $${X_{i}}^{t+1}={X_{i}}^{t}+L.\left({X_{i}}^{t}-\text{g}*\right)$$

where:

${X_{i}}^{t}$: the pollen i or the solution vector$\;{X_{i}}^{\;}$at *t*^*th*^ iteration.

g∗: the current best solution among the current options in the current iteration.

L: The strength of pollination which basically is a step size. In order to imitate the movement of insects over long distances Levy flight distribution is used taking into account that insects move with different distance steps, So L > 0 can be expressed using Levy distribution as in Eq. (9) [12]:

(9) $$L∼\frac{\lambda \;\tau \;\left(\lambda \right).\sin\left(\frac{\pi \lambda }{2}\right)}{\pi }.\frac{1}{s^{1+\lambda }}\left(s\gg 0\right)$$

where τ(λ) is the standard gamma function, and this distribution is valid for large steps (S>>0).

Step 3 If the random value is greater than the probability of switching, start from a uniform distribution. Select *J* and *K* among all other solutions and perform a local pollination. The local pollination and flower constancy can be given as in Eq. (10) [12, 13]:

(10) $${X_{i}}^{t+1}={X_{i}}^{t}+\in .\left({X_{j}}^{t}-{X_{k}}^{t}\right)$$

where ${X_{j}}^{t}$,$\;{X_{k}}^{t}$ are the pollens from different flowers of the same plant species. This essentially simulates the stability of flower in a limited vicinity. If both ${X_{j}}^{t}$and$\;{X_{k}}^{t}$belong to the same species or the same population, this becomes a local random walk if we draw ∈ from a uniform distribution in the domain [0, 1].

**Figure 5:**
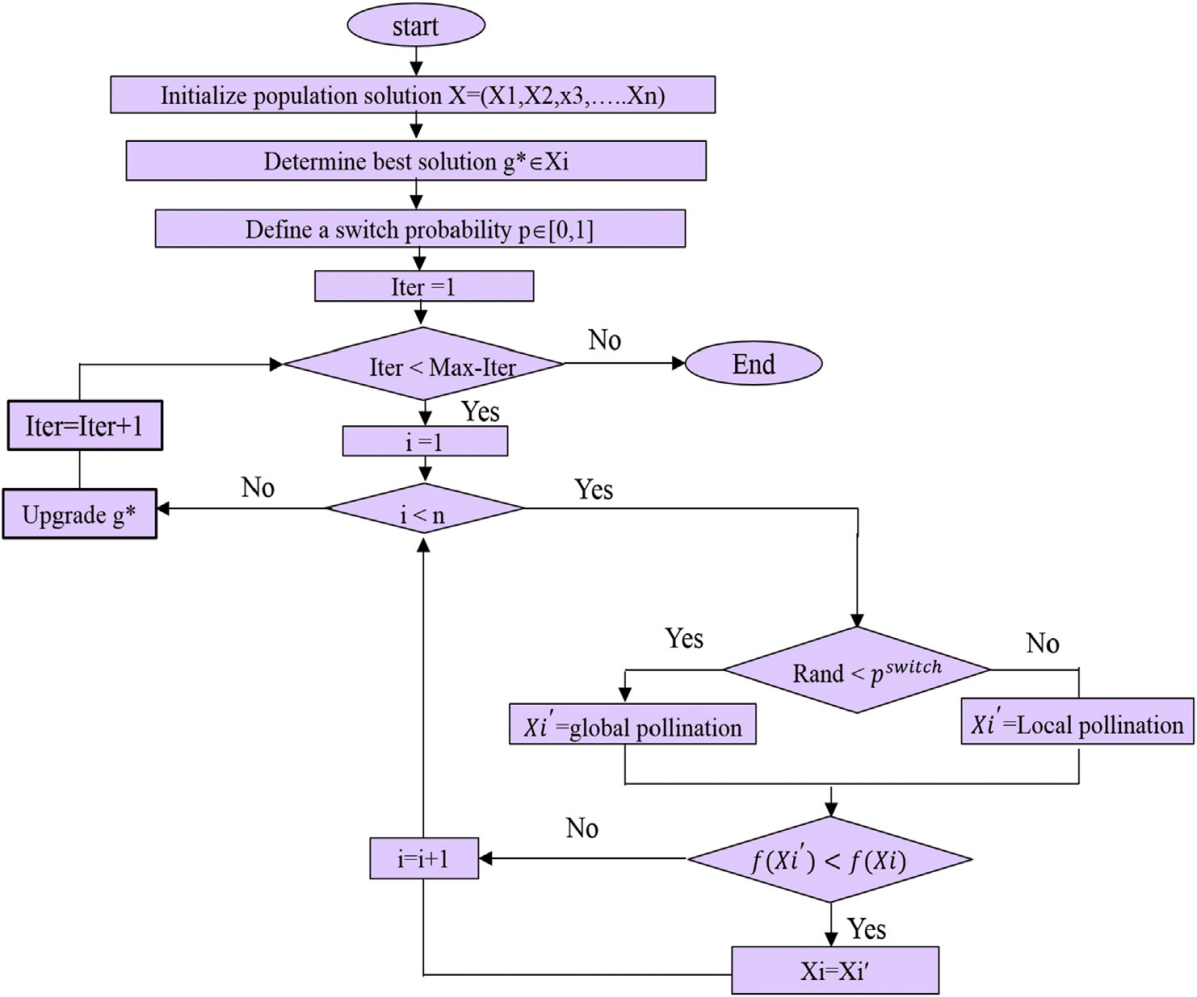
Flow chart of flower pollination algorithm.

Step 4 Finding new solutions for all flowers, where *w*, *r*, *t* are new solutions. If the new solutions are better, replace them in the population then repeat the steps for all members of the population and find the current best solution. The flowchart of flower pollination algorithm is given in figure (5).

## 4 Proposed study

In this study, FPA algorithm is used to obtain optimal P & PI controllers' parameters. Firstly, nine test functions are used to confirm the validity of the proposed algorithm, then the performance of this algorithm is compared with GA, PSO, and GWO algorithms’ performances in terms of time response of the VSD system and values of both THD and RF factors for grid current and DC-link respectively.

The objective function used in this study is the integration of time multiplied be absolute error ITAE that is expressed by Eq. (11):

(11) $$\text{J}\left({\text{ITAE}}_{\;}\right)=\int_{0}^{\infty }t.\left|e\left(t\right)\right|.dt$$

where t is the integration time and e(t) is the error of the studied system [14].

The error e(t) used in objective function equation is the sum of DC-link current error and DC-link voltage error, and it is given by Eq. (12):

(12) $$\text{e}\left(t\right)=\left(V_{0}-ref\left(V_{0}\right)\right)+\left(I_{L}-ref\left(I_{L}\right)\right)$$

The absolute value of the sum of these two errors is multiplied by integration time to form the objective function. The FPA algorithm depends on this objective function in its iterations. When this function reaches its minimum value, it means that the algorithm found the optimal values of controllers' parameters. The smaller the value of the objective function the better the values of the controllers’ parameters.

Two MATLAB scripts were written for both FPA and GA algorithm, then linked with the MATLAB/Simulink model of the studied system. The dynamic performance (rise time and overshoot) and the static performance (stabilization time and steady-state error) were taken into account in the simulation results. The parameters of FPA algorithm are shown in Table 1.

**Table 1:** FPA parameter values.

The parameter	value
Population size (n)	20
Probability of switching (p)	0.8
Number of iterations	150
Number of variables	3
Maximum limits [kp1, kp2, ki]	[40 30 10]
Minimum limits [kp1, kp2, ki]	[0 0 0]

## 5 Results & discussion

Table 2 shows the optimal values of P and PI controllers’ parameters that we obtained using both GA and FPA algorithm.

**Table 2:** Optimal values of P and PI controllers’ parameters.

Flower Pollination Algorithms (FPA)			Genetic Algorithms (GA)		
P controller	PI controller		P controller	PI controller	
Kp	Kp	Ki	Kp	Kp	Ki
30	4	6	20	2	7

**Figure 6:**
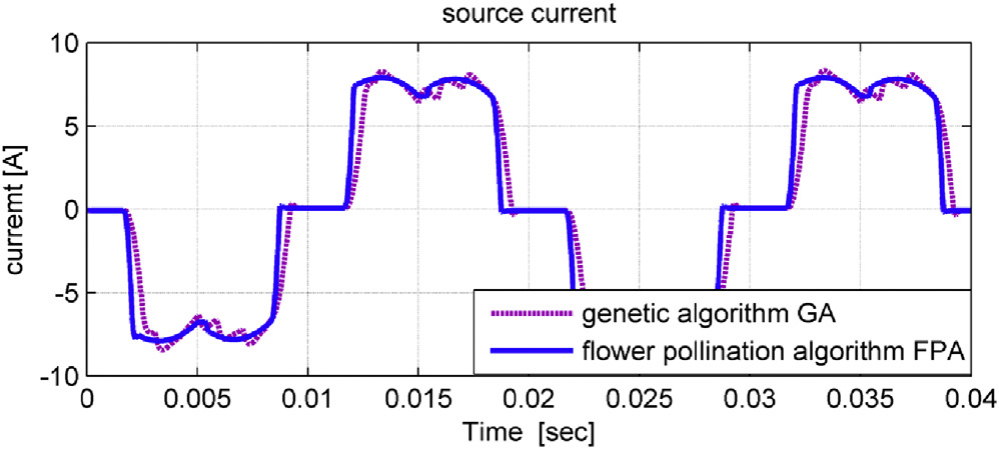
Grid current using both GA &FPA algorithms.

The values obtained by FPA has reduced the total harmonic distortion THD to 27% while the values obtained by GA has reduce THD to 30% which means the source current wave has been improved by using FPA. It is noticed from figure (6) that the source current has less distortion when using FPA than using GA.

**Figure 7:**
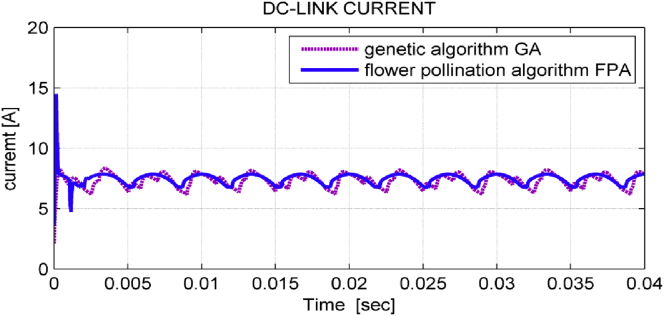
DC-link current in both GA &FPA algorithms.

Figure (7) shows DC-link current when using FPA and GA. It is observed that this current is smoother when using FPA. However, this algorithm gives a greater DC-link current overshoot in comparison with GA.

**Figure 8:**
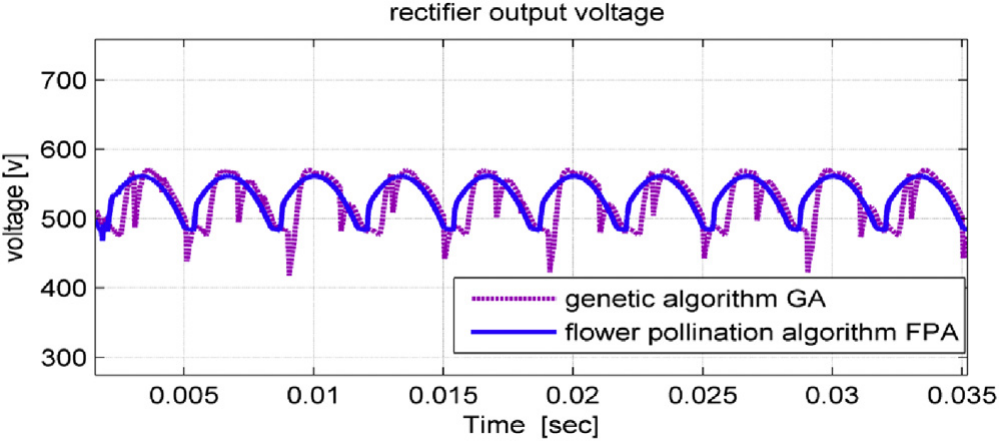
Rectifier output voltage in studied system.

**Figure 9:**
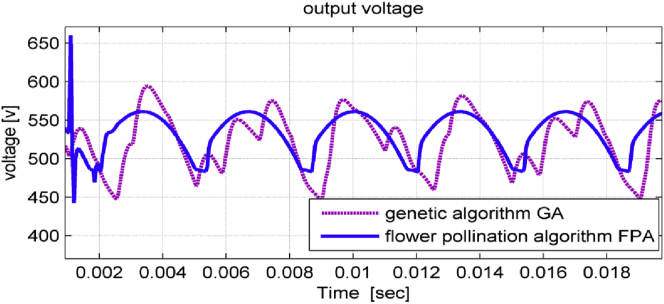
Dc-link voltage (output voltage).

Both the rectifier output voltage and DC-link voltage signals have less ripples when using FPA as shown in Figures 8 and 9 respectively.

**Figure 10:**
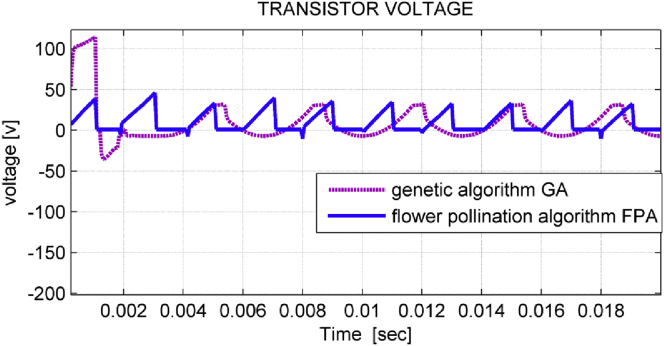
Transistor voltages.

The steady-state response of the reverse voltage on the filter transistors was slightly affected by using FPA algorithm, but the transient response is remarkably improved as shown in figure (10).

Previous researches proposed grey wolf optimization GWO and particle swarm optimization PSO algorithms besides manual tuning to find best controllers’ parameters in Medium power VSD systems [15, 16, 17]. The performances of each algorithm are evaluated based on grid side current total harmonic distortion THD_I_ and DC-link ripple factor RF values in the studied system in addition to the number of iterations *Iter* needed by the algorithm to reach the optimal values [18, 19]. A comparison between these algorithms performances in the VSD system is shown in Table 3.

**Table 3:** VSD system response using different algorithms.

Algorithms	*Iter*	*THD*_*I*_	*RF*
FPA	80	27%	0.16
GA	100	30%	0.2
GWO	90	28%	0.18
PSO	85	28%	0.19
Manual Tuning	-	31%	0.2

As noticed from Table 3, the FPA algorithm outperformed all the other algorithms in terms of reducing grid side current total harmonic distortion *Thd*_*I*_ and dc-link ripple factor RF. In addition, FPA requires 80 iterations to reach the optimal values, which is less than the number of iterations required by other algorithms.

**Figure 11:**
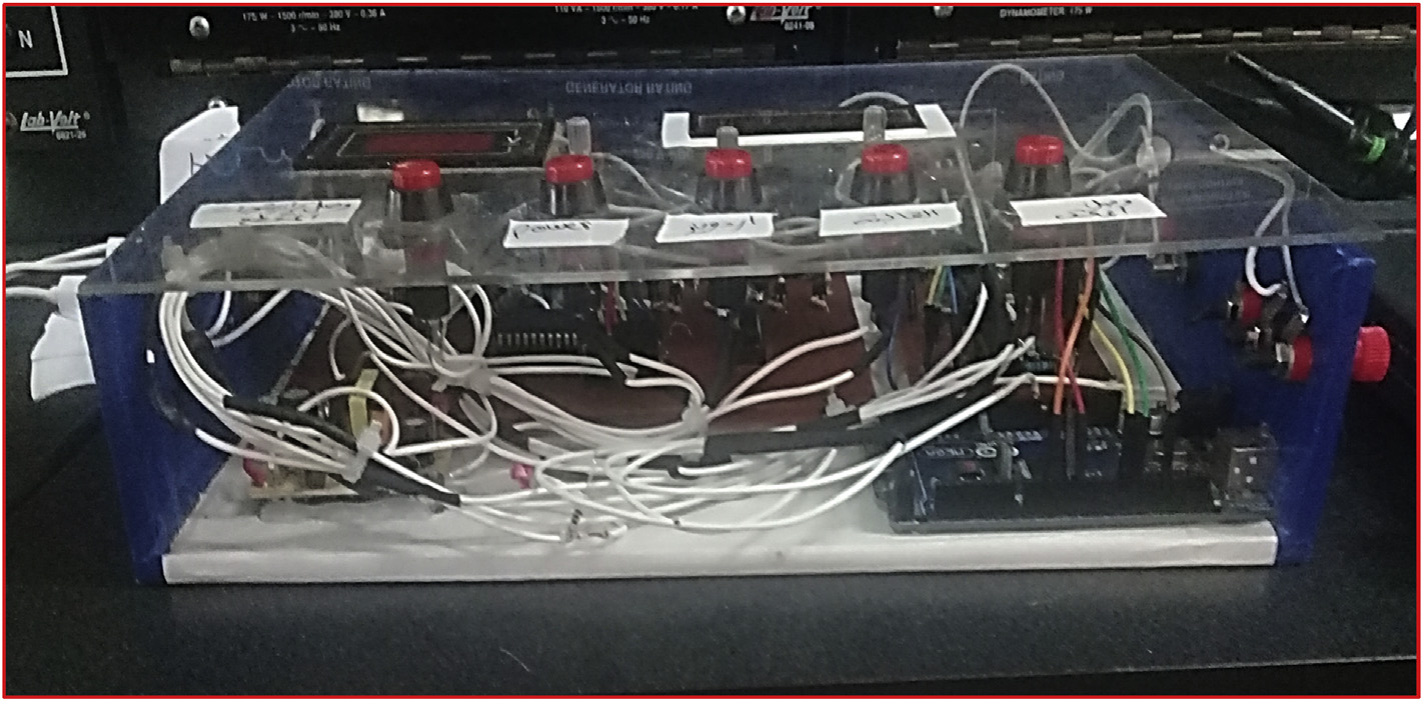
Implemented laboratory model.

In order to confirm the experimental validation of the suggested study, a hardware laboratory model of the studied system has been built to support the simulation results. Figure (11) shows the implemented laboratory model. This model consists of two main parts: control circuit and drive circuit. The drive circuit consists of a three-phase diode bridge rectifier, DC-link, and a four-quadrant chopper integrated with DC-link, while the control circuit includes two micro controllers: A PIC16F877 micro controller used to control chopper transistors and An Arduino mega micro controller used to control inverter transistors.

**Figure 12:**
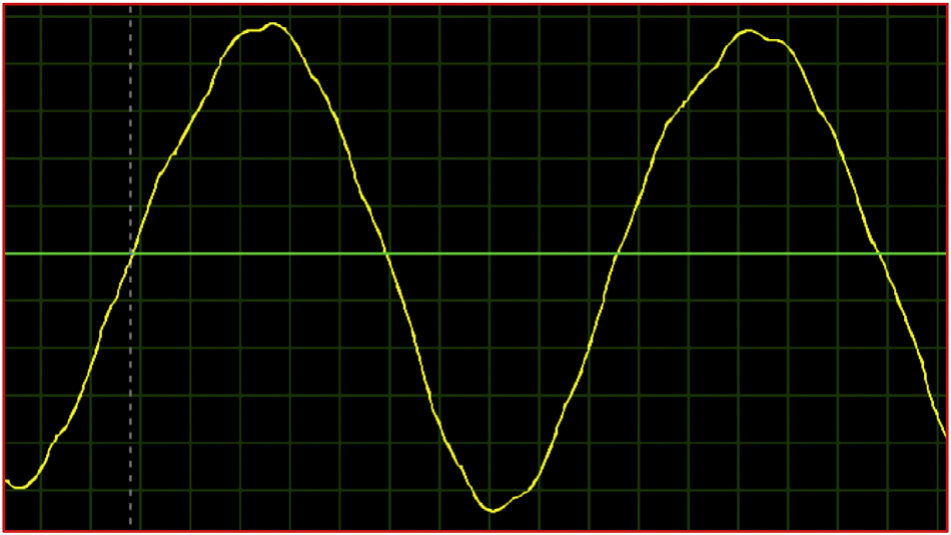
Inverter current at (resistive – inductance) load.

The inverter output current when the system is loaded with (resistive-inductance) load is shown in figure (12). It is notice that this current is approximately sinusoidal with a low total harmonic distortion factor, which illustrates the contribution of the added chopper circuit in improving the quality of the current on the studied system output.

**Figure 13:**
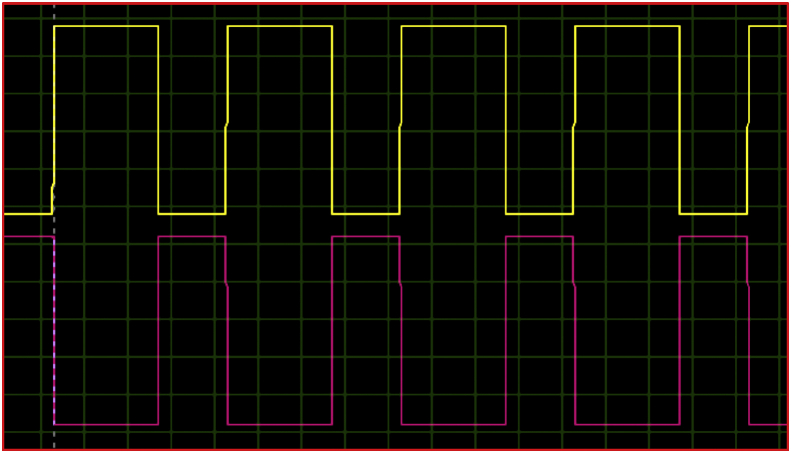
Control signals for chopper transistors.

Figure (13) shows the control signals for the transistors of a single phase of the four -quadrant chopper. We notice from the figure that each opposite transistor operates at complementary gate signals with a very small-time interval between them (dead time).

**Figure 14:**
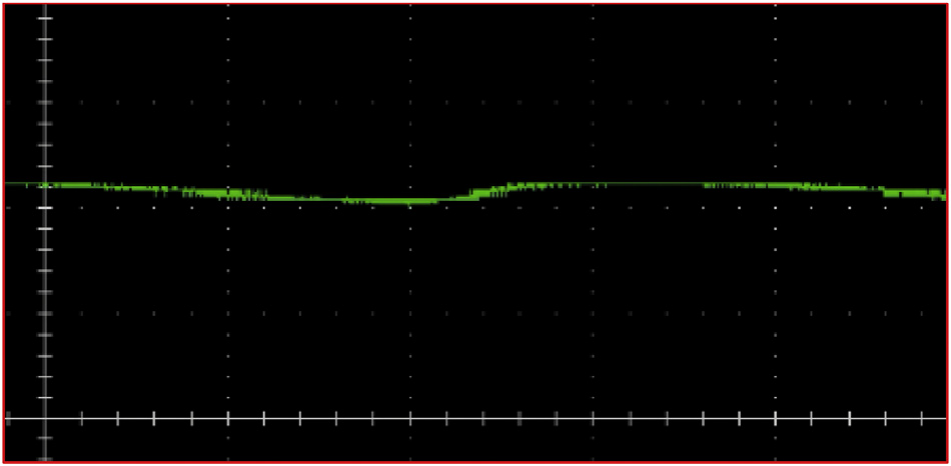
Dc-link voltage.

The response of the implemented system has been studied at a motor power of 4kw. Figure (14) shows the Dc-link voltage.

It is notice that the DC-link voltage has no oscillation and reduced ripples. The ripple factor after adding the chopper circuit is about 16%. The absence of oscillations and the reduction of ripples in the dc-link voltage after adding the chopper circuit, which confirms that the hardware model experimental results are identical to the simulation results and proves the validity of the proposed study.

## 6 Conclusion & recommendation

In this study, both GA & FPA algorithms have been used for parameters tuning of P& PI controllers, these controllers are located in SPWM control circuit that generates the control signals for the filter transistors. The results of the proposed study are as follows:

• FPA algorithm confirmed its superiority against other optimization algorithms.
• The time response of the studied system has been improved.
• The THD% factor of the network current has been reduced to a value of 27%.
• The DC-link current and voltage ripple factor RF has been reduced to value of 0.16%
• FPA-based PI and P controllers have proved to be an efficient technique in generating control signals for filter transistors.

As a future work, we recommend using other optimization algorithms such as Imperialist Competitive Algorithm ICA to achieve the optimal time response for the studied system. We also recommend the use of the FPA algorithm for the optimal selection of inductors and capacitors values in power systems as a future work.

## Declarations

### Author contribution statement

Safwan Nadweh, Ola Khaddam, Ghassan Hayek, Bassam Atieh & Hassan Haes Alhelou: Conceived and designed the experiments; Performed the experiments; Analyzed and interpreted the data; Wrote the paper.

### Funding statement

This research did not receive any specific grant from funding agencies in the public, commercial, or not-for-profit sectors.

### Competing interest statement

The authors declare no conflict of interest.

### Additional information

No additional information is available for this paper.
